# Validation of the ALS Assay in Adult Patients with Culture Confirmed Pulmonary Tuberculosis

**DOI:** 10.1371/journal.pone.0016425

**Published:** 2011-01-21

**Authors:** Rokeya Sultana Rekha, S. M. Mostafa Kamal, Peter Andersen, Zeaur Rahim, Md. Imranul Hoq, Gul Ara, Jan Andersson, David Sack, Rubhana Raqib

**Affiliations:** 1 International Centre for Diarrhoeal Diseases Research, Bangladesh (ICDDR,B), Dhaka, Bangladesh; 2 National Institute of Diseases of Chest and Hospital (NIDCH), Dhaka, Bangladesh; 3 Statum Serum Institute, Copenhagen, Denmark; 4 Department of Medicine, Karolinska Institutet, Karolinska University Hospital, Huddinge, Sweden; McGill University, Canada

## Abstract

**Background:**

We have earlier shown that *Bacille Calmette-Guérin* (BCG) vaccine-specific IgG Antibodies in Lymphocyte Supernatant (ALS) can be used for diagnosis of active tuberculosis (TB) in adults and children.

**Methodology/Principal Findings:**

The ALS method was validated in a larger cohort (n = 212) of patients with suspicion of pulmonary TB using multiple antigens (BCG, LAM, TB15.3, TB51A, CFP10-ESAT6-A, CFP, CW) from *Mycobacterium tuberculosis*. The sensitivity and specificity of the ALS assay was calculated using non-TB patients as controls. The sensitivity and the specificity were highest with BCG vaccine (90% and 88% respectively) followed by LAM (89% and 87% respectively). Simultaneous assessment of multiple antigen-specific antibodies increased sensitivity (91%) and specificity (88%). Using higher lymphocyte count in smaller volume of culture media increased detection and reduced the assay duration to ∼30 hrs. Twenty one patients with clinical findings strongly suggestive of TB finally diagnosed as non-TB patients were positive by the ALS assay, of which 9 (43%) were positive for 7 antigens and 19 (90%) for at least 3 antigens.

**Conclusions/Significance:**

Our findings show that simultaneous detection of antigens improves the diagnostic potential of the ALS assay; the modified method increases sensitivity and can provide results in <48 hours, and enable detection of some cases of pulmonary TB that are not detectable by standard methods.

## Introduction

Tuberculosis (TB) caused by *Mycobacterium tuberculosis* (MTB) remains a major global public health problem which is responsible for over 3 million deaths annually. Recent analysis of global burden of TB revealed that Bangladesh ranks 6^th^ on the list of 22 highest burden TB countries in the world [1]. These 22 countries collectively account for 80% of incident TB cases globally. An efficient TB control program requires early and accurate diagnosis and treatment of patients with active pulmonary TB. A major obstacle to early treatment of TB is the lack of rapid accurate diagnostic methods that can be applied in low-income areas. Diagnosis of active mycobacterial infections in resource-constrained settings is primarily based on clinical examination, radiological findings, and identification of acid-fast bacilli in sputum by smear microscopy. Microbiological culture requires specialized laboratories and is done rarely.

A serological test detecting circulating antibodies against specific mycobacterial antigens in serum is an attractive approach for the diagnosis of TB due to its speed and relatively simple technology. However, serological tests have been hampered by decreased sensitivity and cross-reactivity with other mycobacteria, and have relatively limited usefulness in the diagnosis of TB in TB endemic countries [2], [3], [4], [5]. Additionally, there is great variability in specific antibody expression in different subjects [6] and antigen composition of *M. tuberculosis* bacilli recognized by antibodies also varies with stages of infection and disease progression [7]. Recent systematic reviews of commercial serological tests showed that, commercial tests vary widely in performance [3], [8]. The sensitivity is higher in smear positive than smear-negative samples; specificity is higher in healthy volunteers than in non-TB suspects where TB was part of the differential diagnosis [3]. Furthermore, serological methods have low discriminatory power differentiating latent from active TB infections in endemic regions [9]. There is a need for methods to accurately diagnose and discriminate between latent and active TB infections in endemic regions.

We have previously shown detection of antibody in lymphocyte supernatants (ALS) is a rapid diagnostic method for the identification of patients with active pulmonary TB in adults [10], [11] and in pediatric patients [12] using BCG vaccine as an antigen. Our aim was to further validate the ALS method in the diagnosis of pulmonary TB among well-characterized patients with symptomatic respiratory diseases and healthy controls, to evaluate TB-specific antigens other than BCG in the assay, and to explore methods to provide results more rapidly. The patients were carefully screened and diagnosed according to the standardized protocol provided by the World Health Organization for TB Specimen Bank project. Multiple TB antigens were used singly and in combination for evaluation in the ALS assay.

## Results

### Characteristics of study participants

Two hundred and twelve patients (median of age, 25 years; range, 18 to 70 years) with suspected pulmonary TB were recruited from the NIDCH. The demographical characteristics are given in Table 1. Patients, especially those who were smear negative, culture negative and treated for TB based on clinical and radiographic evaluation (category 3, n = 9) and those who were smear negative, culture negative and were not treated for TB (category 4 and 9) were followed for 8–12 weeks. The day of enrollment was considered day 1. Among the category 3 TB index cases, 6 were lost to follow-up at the 2-month time point. These patients were being treated with anti-TB therapy and assessment of clinical and radiological improvement for confirming TB disease at 2 months follow-up could not be ascertained, although improvement was noticed at the 1 month follow-up. Four isolates were identified as nontuberculous *Mycobacterium* (NTM) though no species identification was done in the study. For calculation of sensitivity and specificity of the ALS method, category-3 (n = 9) group was not included since TB disease in this group was confirmed by an indirect method only i.e. response to anti-TB therapy where 6 were lost to follow-up. Among the category 9 patients most cases were later diagnosed to have bronchiectasis, chronic obstructive pulmonary diseases, bronchitis, lung cancer and lung abscess. About 30 patients remain indeterminate.

**Table 1:** Characteristics of patients suspected of having active pulmonary TB and healthy controls.

Characteristics	TB patients	Non-TB patients	Healthy controls
Total Number	112	100	25
	Cat-1 = 104	Cat-4 = 10	
	Cat-2 = 8	Cat-9 = 90	
Median age, years	24 (18–70)	26 (18–68)	26 (20–50)
Male∶ Female	64∶48	54∶46	15∶10
BCG vaccination	41 (36.6%)	42 (42.0%)	25 (100%)
Known history of TB contact	59 (52.7%)	61 (61.0%)	0
Previous history of TB	11 (9.8%)	7 (7.0%)	0
Occupation			
Garments workers	42 (37.5%)	33 (33.0%)	-
Daily Laborers	20 (17.9%)	17 (17.0%)	-
Housewives	15 (13.4%)	18 (18.0%)	-
Students	11 (9.8%)	4 (4.0%)	9 (36.0%)
Service holders	6 (5.4%)	7 (7.0%)	16 (64.0%)
Others	18 (16.0%)	21 (21.0%)	-

All TB patients received the standard treatment by directly observed treatment short course (DOTS). BCG vaccination status was confirmed by obtaining medical history from each patient and inspection of visible scars that typically forms after vaccination with *M. bovis* BCG. None of the patients were positive for HIV infection. A total of twenty five healthy controls (median of age 26 years; range, 22 to 50 years) were enrolled in the study. Eight out of twenty five healthy subjects had MT a skin induration ≥10 mm.

### The micro-ALS method using higher PBMC concentrations gives higher BCG-specific IgG responses in shorter time

**Figure 1:**
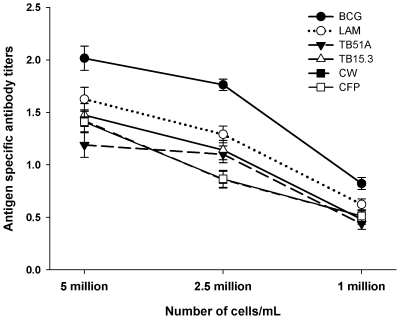
Antigen specific antibody titers against 6 different antigens at various cell concentrations in the micro-ALS assay. Higher concentrations of peripheral blood mononuclear cells produced higher antigen-specific IgG responses. BCG-specific IgG antibody titers were highest followed by LAM-specific IgG antibody levels; antibody responses against CFP, TB51A, TB15.3 and CW were similar.

Culture supernatants from different concentrations of cell suspensions showed that higher concentrations of PBMCs (e.g. 5 million) gave higher specific IgG titers for all antigens in TB patients at a given time point compared to low cell count (2.5 or 1 million) (Figure 1). This was done in a sub-sample of 30 patients (smear and culture positive). At any given cell concentration, antigen-specific antibody titers were highest against BCG followed by LAM; antibody responses against CFP, TB51A, TB15.3 and CW were similar.

Using the micro-ALS method with elevated cell concentration (10×10^6^ PBMC) in 400 µl culture media, antibody titers were already high at 24 hrs (mean titers ± standard deviation, 0.88±0.21), and there was only a small increase in antibody responses after 48 hr (0.99±0.19) in patients. Thus, the assay could be shortened by culturing the PBMC overnight for 16–18 hours followed by 7 additional hours to run the ELISA assay (total duration of assay ∼30 hours). Supernatant of 10×10^6^ PBMC from healthy controls showed titers lower than the cut-off and there was a minimal increase from 24 (0.17±0.07) to 48 hrs (0.20±0.09). About 3–4 ml blood was found to be adequate for this micro-ALS method.

### Diagnostic performance of the ALS assay for single antigens

The intra-assay variation (Coefficient of Variance) was 2.41%. Repeated freeze-thaw of supernatant interferes with the assay greatly; thus multiple aliquot of supernatant was collected. Using new aliquot for the assay gave inter-assay variation of 3.59%, while use of freeze thawed supernatant gave a variation of 6.89%. Receiver-operator characteristic curves were constructed from the ALS responses to all antigens, comparing TB patients with non-TB patients. The selection of the best cutoff point was based on the level at which the accuracy was maximum for each antigen. The cut-off values for all antigens were very close (0.408 to 0.423) except for CFP10-ESAT6-A (0.397). The sensitivity, the specificity, positive/negative predictive values and positive/negative likelihood ratios of the ALS assay was calculated in comparison to non-TB group (Table 2). The assay using BCG-vaccine as the antigen showed the highest combination of specificity and sensitivity (Table 2); the sensitivity was somewhat lower for RD1 antigens TB15.3 and TB51A although the specificity was similar. The specificity of the fusion protein CFP10-ESAT6-A was about 86% and the sensitivity was lower compared to BCG, LAM, TB15.3 or TB51A. The negative predictive value was highest for BCG (91%) followed by LAM and TB51A (90%) and TB15.3 (89%) (Table 2). The positive likelihood ratio (LR+) was good (7.50 and 6.85) for BCG and LAM and suggested conclusive increase in the likelihood of disease, while for TB51A it was moderate increase (6.14) in the likelihood of disease. For TB15.3 and CFP10-ESAT6-A, the LR+ value depicted small increase in the likelihood of disease. The negative likelihood ratio (LR−) for BCG and LAM was similar and indicated moderate decrease in the likelihood of disease (0.1–0.2). The LR- for TB15.3, TB51A and CFP10-ESAT6-A indicated a small decrease in the likelihood of disease (0.21–0.5).

**Table 2:** Diagnostic performances of the ALS assay by using single TB specific antigens and simultaneous determination of these antigens in various combinations.

TB Antigens	Sensitivity (95% CI)	Specificity (95% CI)	PPV	NPV	LR−	LR+
BCG, n = 212	90% (82%–94%)	88% (81%–93%)	87%	91%	0.11	7.50
LAM, n = 210	89% (83%–93%)	87% (79%–92%)	85%	90%	0.13	6.85
TB15.3, n = 210	85% (77%–91%)	86% (78%–91%)	88%	89%	0.17	6.07
TB51A, n = 207	86% (78%–91%)	86% (78%–91%)	88%	90%	0.16	6.14
CFP10-ESAT6-A, n = 108	73% (67%–81%)	86% (76%–91%)	86%	88%	0.31	5.21
CW, n = 209	86% (77%–91%)	84% (76%–90%)	83%	87%	0.17	5.38
CFP, n = 207	82% (74%–88%)	83% (74%–89%)	83%	82%	0.22	4.82
BCG and LAM	91% (84%–95%)	89% (82%–94%)	86%	91%	0.10	8.27
BCG and TB51A	88% (80%–93%)	87% (79%–92%)	88%	91%	0.14	6.77
TB51A and TB15.3	86% (78%–91%)	86% (78%–91%)	86%	88%	0.16	6.14
TB51A, TB15.3 and LAM	87% (79%–92%)	88% (80%–93%)	86%	89%	0.15	7.25
BCG, LAM and TB51A	89% (82%–94%)	87% (79%–92%)	87%	91%	0.13	6.85
BCG, CFP and LAM	91% (83%–95%)	86% (79%–92%)	85%	92%	0.10	6.50
BCG, CFP, LAM and TB51A	89% (81%–94%)	86% (78%–91%)	86%	92%	0.13	6.36
BCG, CFP, LAM, TB51A and TB15.3	89% (81%–94%)	87% (79%–92%)	85%	91%	0.13	6.85
BCG, CFP, LAM, TB51A, TB15.3 and CFP10-ESAT6-A	89% (81%–94%)	86% (79%–92%)	86%	90%	0.13	6.36
BCG, CW, CFP, CFP10-ESAT6-A, LAM, TB51A and TB15.3	91% (83%–95%)	88% (81%–93%)	86%	90%	0.10	7.58

BCG vaccine- glutamate-BCG vaccine for intradermal use, lot 1861, Japan BCG Laboratories, Japan; CFP10-ESAT6-A- fusion protein of Culture filtrate protein-10 and Early secretory antigenic target-6; TB51A, TB15.3 antigens from RD1 region; CFP- culture filtrate proteins and CW− cell wall fraction from *M. tuberculosis* H37Rv and clinical isolate CSU93; LAM− lipoarabinomannan of *M. tuberculosis* H37Rv. ALS titers are given from 48 hrs culture. Receiver-operator characteristic curves were constructed from the ALS responses to all antigens, comparing TB patients with non-TB patients to select the best cutoff point at which the accuracy was maximum for each antigen. The cut-off values for all antigens were very close (0.408 to 0.423) except for CFP10-ESAT6-A (0.397). All patients could not be tested for all 7 antigens because in some cases we did not have enough ALS supernatant.

When the sensitivity, the specificity, negative/positive predictive values as well as LR+ and LR- of the ALS assay were calculated in comparison to healthy controls, all these parameters substantially increased as expected (data not shown).

### Diagnostic performance of the ALS assay increases with simultaneous detection of multiple antigen-specific antibodies

The sensitivity and specificity of the ALS assay increased when antibodies specific against multiple antigens were determined simultaneously. Thus, a specimen found to be positive for any of the 7 antigens in the assay was considered to be positive. This led to a slight increase in sensitivity, specificity and LR+ of the assay and a decrease in LR−. The combination of all 7 antigens gave sensitivity, specificity and the LR+ of 91%, 88% and 7.58 respectively while a combination of 3 antigens also showed values close to this, 91%, 86% and 6.50 respectively (Table 2). The best combination however, was BCG plus LAM that gave the sensitivity, the specificity and LR+ of 91%, 89% and 8.27 respectively.

### Nontuberculous *Mycobacterium* infection and ALS titers

We found 4 cases of non-tuberculous *Mycobacterium* (NTM) infections, one of these patients showed ALS titers higher than the cut-off against CFP only (0.75), one patient had higher titers against CFP (0.52), CW (0.44), LAM (0.47), CFP-10-ESAT-6A (0.55) but not against BCG (0.36). Two patients did not show any response against the 6 antigens.

### Non-TB patients with ALS titers higher than the cutoff

There were specimens from twenty one patients belonging to categories 4 (n = 1) and 9 (n = 20) whose ALS responses were higher than the cutoff. Specimens from 9 (43%) of these patients responded to ≥6 TB antigens, and a specimen from only one patient was positive for only one antigen (Table 3). All of these patients had more than six typical symptoms, 43% had haemoptysis and 67% had history of contact with active TB.

**Table 3:** Profiles of non-TB and category 3 patients with ALS titers higher than the cutoff and TB patients with ALS titers lower than the cutoff.

Category 4 and 9 patients with ALS titers higher than the cutoff								
Positive for	No. of patients	Previous TB	History of TB contact	BCG given	Abnormal CXR	Typical Symptoms present		Haemoptysis
						At least 6	more than 6	
≥6 Antigens	9 (43%)	0 (0%)	8 (89%)	2 (22%)	6 (67%)	0 (0%)	9 (100%)	5 (56%)
≤5 Antigens	12 (57%)	0 (0%)	6 (50%)	4 (33%)	11 (92%)	2 (17%)	10(83%)	4 (33%)
Total	21 (100%)	0 (0%)	14 (67%)	9 (28%)	17 (81%)	2 (10%)	14 (90%)	9 (43%)
Positive for			Category 3 patients with ALS titers higher than the cutoff					
≥6 Antigens	3 (33%)	1 (33%)	1 (33%)	2 (67%)	3 (100%)	0 (0%)	2 (67%)	1 (33%)
≤5 Antigens	6 (67%)	2 (33%)	3 (50%)	3 (50%)	6 (100%)	1 (17%)	3 (50%)	3 (50%)
Total	9 (100%)	3 (33%)	4 (44%)	5 (55%)	9 (100%)	1 (11%)	5 (55%)	4 (44%)
Negative for	Category 1 and 2 TB patients with ALS titers lower than the cutoff							
≥6 Antigens	10 (34%)	1 (10%)	6 (60%)	5 (50%)	10(100%)	1 (10%)	9 (90%)	7 (70%)
≤5 Antigens	19 (66%)	3 (16%)	5 (26%)	9 (47%)	19 (100%)	1 (5%)	18 (95%)	7 (37%)
Total	29 (100%)	4 (14%)	11 (38%)	14 (48%)	29 (100%)	2 (7%)	27 (93%)	14 (48%)

TB− tuberculosis; BCG− *Bacille Calmette-Guérin* vaccine; CXR, X-ray of the chest.

### TB patients with ALS titers lower than the cut-off

Twenty nine of one hundred and twelve culture and/or smear positive TB patients (categories 1 and 2) had ALS titers lower than the cut-off (0.42) for one or more antigens. Ten of one hundred and twelve patients (8.9%) were negative for ≥6 antigens and 19 (17%) were non-responding to at least one antigen. Ten patients who were negative for ≥6 antigens were smear and culture positive. Nine of these ten patients had presence of more than 6 typical symptoms while one had at least 6 symptoms, six had a history of contact with active TB, seven had haemoptysis and all ten had abnormal chest radiograph (Table 3).

### Comparison of ALS assay performance between smear positive and smear negative cases

Specific antibody levels to single antigens LAM and TB51A in AFB+ TB patients were significantly higher than AFB− TB patients (P<0.02 and P<0.02 respectively). No significant differences were observed between the two patient groups with respect to other antigens. When simultaneous detection of all TB antigen-specific antibodies were considered, the levels of total specific antibodies were found to be significantly higher in the supernatant of the AFB smear-positive (AFB+; category 1) TB patients than the AFB smear negative (AFB−; category 2) TB patients (P<0.001) (Table 4).

**Table 4:** Comparison of performance of the ALS assay between smear positive (C-1) and smear negative (C-2) TB cases.

TB Antigens		Sensitivity (95% CI)	Specificity (95% CI)	LR−	LR+
BCG	AFB+	91% (83%–95%)	88% (81%–93%)	0.10	7.58
	AFB−	88% (80%–93%)	88% (80%–93%)	0.14	7.33
LAM	AFB+	92% (84%–96%)	87% (80%–93%)	0.09	7.08
	AFB−	86% (78%–92%)	87% (79%–92%)	0.16	6.62
TB15.3	AFB+	87% (79%–92%)	86% (78%–92%)	0.15	6.21
	AFB−	71% (67%–82%)	86% (74%–90%)	0.34	5.07
TB51A	AFB+	88% (80%–93%)	86% (78%–92%)	0.14	6.29
	AFB−	79% (71%–86%)	86% (77%–91%)	0.24	5.64
CW	AFB+	89% (81%–93%)	84% (77%–90%)	0.13	5.56
	AFB−	79% (71%–87%)	84% (75%–89%)	0.25	4.94
CFP	AFB+	88% (80%–93%)	83% (75%–90%)	0.14	5.18
	AFB−	76% (69%–84%)	83% (74%–89%)	0.29	4.47
BCG and LAM	AFB+	93% (86%–96%)	89% (82%–94%)	0.08	8.45
	AFB−	87% (79%–92%)	89% (81%–94%)	0.15	7.91
All Ag	AFB+	92% (84%–96%)	88% (81%–93%)	0.09	7.67
	AFB−	88% (80%–93%)	88% (80%–93%)	0.14	7.33

BCG vaccine- glutamate-BCG vaccine for intradermal use, lot 1861, Japan BCG Laboratories, Japan; CFP10-ESAT6-A- fusion protein of Culture filtrate protein-10 and Early secretory antigenic target-6; TB51A, TB15.3 antigens from RD1 region; CFP− culture filtrate proteins and CW− cell wall fraction from *M. tuberculosis* H37Rv and clinical isolate CSU93; LAM− lipoarabinomannan of M. tuberculosis H37Rv. AFB+, sputum AFB smear positive; AFB−, sputum AFB smear negative. ALS titers are given from 48 hrs culture. The cut-off values for all antigens were very close (0.408 to 0.423) except for CFP10-ESAT6-A (0.397).

The sensitivity of the BCG-specific ALS assay in AFB+ patients decreased slightly from 91% to 88% in AFB− patients without compromising the specificity (88%) (Table 4). The sensitivity of the LAM-specific ALS assay in AFB+ patients decreased from 92% to 87% in AFB− patients, the specificity remained unchanged at 87%. Similar data were obtained for the rest of the antigens; the specificity did not change but the sensitivity decreased to some extent (Table 4).

## Discussion

The study showed that compared to single antigens, simultaneous detection of multiple antigens increased sensitivity, specificity and positive likelihood ratio of the ALS assay in the diagnosis of active TB. BCG vaccine and LAM were the single antigens that gave highest combination of sensitivity and specificity. The micro-ALS method shortened the duration of the assay and increased the possibility of detecting borderline cases.

We have earlier applied the ALS assay in diagnosing active TB disease in adults, in their household contacts and in pediatric patients [10], [11], [12] where BCG vaccine was used as the coating antigen for ELISA. In this study multiple antigens were used to identify suitable *Mycobacterium tuberculosis* specific antigens that might be better compared to the BCG vaccine. The expression of proteins encoded on the region of difference (RD-1) domain of the genome (e.g. ESAT-6, CFP-10, TB15.3 and TB51A) is known to be absent in BCG substrains and many environmental mycobacteria [13], [14], [15]. We found that BCG vaccine gave the highest accuracy and precision compared to other single antigens. The RD1 antigens TB15.3, TB51A and fusion protein CFP10-ESAT6-A had high specificity but the sensitivity was lower than for BCG. BCG vaccine strain being a large molecule consisting of diverse types of antigens, including lipids and proteins may have provided a broader range of antigenic epitopes for capturing antibodies from circulating plasma cells [14]. Again, simultaneous determination of multiple antigen specific antibodies also increased the sensitivity and specificity of the ALS assay and could enhance the possibility of case detection. This was in agreement with the recent observations of other researchers showing combination of antigens to be more effective in serological assays for diagnosis of TB with increased sensitivity and specificity compared to single antigens [16], [17]. Interestingly, the accuracy of the ALS method using simultaneous determination of BCG and LAM responses was higher than the combination of 6 antigens. The specificity of the ALS assay was substantially higher when healthy controls were considered instead of non-TB controls (data not shown).

The use of higher PBMC concentration in a smaller volume of culture media in the micro-ALS method allowed for increased concentration of released antibodies in the supernatant thus permitting a shorter period of cell culture and increased detection. The micro-ALS method also had the potential to identify borderline TB cases that might otherwise be missed due to lower production of specific antibodies by low cell numbers. Severe to moderately ill TB patients exhibit significantly lower cell count per ml of blood compared to healthy subjects (personal observation). More importantly pediatric patients produce lower amounts of antibodies in serum compared to adults [18], [19], [20] and have a greater risk of being ruled out for TB in serological assays. In elderly and immunocompromised patients (e.g. having HIV and renal diseases) with TB as a co-infection, definitive diagnosis becomes challenging due to nonspecific clinical and radiological signs. Serological and interferon gamma release assays (IGRAs) in these patients show suboptimal sensitivity [21], [22], [23], [24], [25] and cannot differentiate between disease and infection. Nucleic acid amplification tests (NAAT) methods are also less sensitive in immunocompromised patients and in paucibacillary TB [26]. The micro-ALS method thus appears promising and may have the potential to be more sensitive in diagnosing TB in immunosuppressed patients.

The positive ALS results for several of the antigens in several patients who had negative smears and cultures, but symptoms consistent with TB suggests that the ALS assay may detect cases of active TB which are not being identified using standard methods. Among the patients in category 4 and 9, the fact that 43% of these patients being positive for ≥6 antigens and 90% for at least 3 antigens suggest that these were true TB cases with typical symptoms in whom TB disease was initially suspected and subsequently ruled out due to negative culture and indeterminate CXR. Although mycobacterial culture is considered the gold standard in TB diagnosis sputum culture is not a very sensitive method, all pulmonary TB cases do not always become sputum culture positive. Thus, possibility of some degree of misclassification of disease remains. Further evaluation of such patients is needed to determine if these patients represent false positive ALS results or false negative culture results.

Among the culture confirmed TB cases, 10 patients were ALS negative for all antigens while the rest responded to at least 1 or more antigens. These false negative findings suggest that further modifications of the ALS assay are needed to improve sensitivity. AFB+ patients had significantly higher levels of antigen-specific IgG titers compared to AFB− patients which has also been seen with serological methods [17] and might be attributed to bacterial burden, levels of antigens, and the stage of disease [7], [9].

There are a number of limitations of the study. ESAT-6 and CFP10 antigens are absent from BCG strains and from most environmental mycobacteria, with the exception of *M. kansasii, M. szulgai, M. marinum* and *M. gordonae* [27]. Our findings indicate that patients with the above NTM infections may be misdiagnosed as *M. tuberculosis* cases by the ALS assay depending on the selection of antigens used and thereby may affect the diagnostic specificity to some extent. The classification of Category 9 patients as non-TB who had high ALS titers and whose conditions worsen or remained unchanged after 2 months of anti-TB therapy was doubtful. It may be possible that some of these patients were infected with MDR TB that were paucibacillary in nature, consequently in absence of a positive sputum culture the diagnosis could not be confirmed. The Mantoux Test showed 8 healthy subjects to have latent TB, none of whom showed ALS-IgG titers higher than the cut-off (against BCG) suggesting that ALS assay does not detect latent TB. However, we did not apply Quantiferon method as a confirmatory/complimentary test for exclusion of latent TB in them.

In conclusion, the findings show that simultaneous determination of at least two antigens, LAM and BCG and application of the micro-ALS method improves diagnostic accuracy of the ALS assay and reduces the assay time considerably. Together with consideration of key clinical characteristics, it can support rapid detection of TB cases, to identify false negative cases and compliment other techniques e.g. IGRA in differentiating latent TB from active TB infection and enable correct judgment on appropriate anti-TB treatment. Further work is ongoing to evaluate the performance of ALS assay in the diagnosis of TB in HIV infected patients and in extra pulmonary TB.

## Materials and Methods

### Ethics Statement

The study was approved by the Ethical Review Committee of the International Center for Diarrheal Diseases Research, Bangladesh (ICDDR,B). All patients gave their written informed consent.

### Study subjects and sampling

Adult patients with suspected pulmonary TB who attended the National Institute of Diseases of the Chest and Hospital (NIDCH) in Dhaka, Bangladesh, were prospectively studied. This study was part of the WHO/TDR Tuberculosis Specimen Bank activity [28] in which blood, sputum/saliva and urine specimens were collected from well-characterized TB patients. All specimens were collected and processed using a standardized protocol provided by the WHO and was accompanied by complete clinical information, including the HIV status. HIV testing was done by standard serology. The diagnosis of TB was established by the clinical presentation, chest radiograph examination, and sputum smear and culture positivity as described in case definition. The typical symptoms included persistent cough (≥ three weeks), fever, malaise, recent weight loss, night sweats, contact with active case (intimate or household contact), hemoptysis, chest pain– pleuritic or otherwise and loss of appetite. Healthy laboratory personnel with no known exposure to *M. tuberculosis* were selected as healthy control subjects. These healthy subjects underwent Mantoux test after blood was collected for the ALS assay, since performance of Mantoux test within the past 30 days influences the ALS test (data not shown). The study was approved by the ethical review committee of International Centre for Diarrhoeal Disease Research, Bangladesh (ICDDR,B). Signed informed consent was obtained from each study subject according to the guidelines of the Institutional Review Board of the Centre.

### Case definitions

Patients with two sputum smears positive for acid-fast bacilli (AFB) and at least 1 culture positive on solid media are considered Category 1 TB patients. Category 2 is a patient with 2 negative smears and at least 1 culture positive and abnormal chest x-ray (CXR). Category 3 is a patient with 2 negative smears and cultures, clinical symptoms, radiographic abnormalities consistent with active PTB, no response to broad spectrum non-TB antibiotics with clinical and radiographic improvement after 3 months of a full course of anti-TB chemotherapy, a decision taken by the clinician. Category 4 patients have 2 negative smears and cultures on initial assessment; CXR is irrelevant and are still negative in smear and culture at 2–3 months follow-up; Category 4 is thus non-TB. Category 9 patients are indeterminate cases with a combination of results not matching categories 1–4 and in whom disease diagnosis may or may not be confirmed.

### Specimens

From each patient sputum samples were collected twice on consecutive days for acid-fast bacilli (AFB) staining. For isolating *Mycobacterium* spp., sputum samples were cultured on solid (Lowenstein-Jensen slants) (L–J) as well as in liquid (manual MGIT, Becton Dickenson, USA) systems following standard procedures. Inoculated L–J slants were weekly checked for characteristic growth of mycobacterial colonies until 8 weeks. Sputum was considered negative when there is no characteristic growth of *Mycobacterium* on L–J slant within 8^th^ weeks. Similarly, inoculated MGIT tubes were also checked for positive fluorescence weekly until 8^th^ weeks. Part of palette (from 500 microliter broth) of fluorescence positive MGIT tube was stained for AFB. When positive, the remaining part of the pellet was inoculated on L–J slant for isolation of *Mycobacterium* spp. A culture was confirmed *M. tuberculosis* based on three biochemical tests: niacin (positive), nitrate (positive) and P-nitro benzoic acid (sensitive). Any deviation from these tests was considered as non-tuberculous *Mycobacterium* [29]. Positive cultures were inoculated for drug sensitivity testing. Blood samples were collected from each patient at enrollment and from healthy subjects for the ALS assay.

### The ALS assay

Peripheral blood mononuclear cells (PBMCs) were separated from blood on Ficoll-Paque by differential centrifugation and suspended in 24-well tissue culture plates (Costar, Cambridge, MA) in tissue culture medium. PBMCs at a cell concentration of (1×10^6^ cells/ml) were incubated at 37°C with 5% CO_2_ and culture supernatants were collected after 48 hrs and stored at −70°C until used for measurement of immunoglobulin G (IgG) titers. Polystyrene microtiter plates (MaxiSorp, Nunc) were coated with TB specific antigens (described below) in carbonate buffer (0.1 M sodium bicarbonate and 5 mM magnesium chloride [pH 9.8]) and incubated overnight at 4°C. After washing, the plates were incubated first with 10% fetal bovine serum in phosphate-buffered saline (pH 7.2) and then with lymphocyte supernatants, each for 2 h at 37°C, with intermittent washing. Horseradish peroxidase-conjugated rabbit anti-human IgG was added and incubated for 2 h at room temperature. Plates were developed with the substrate *O*-phenylenediamine and the optical density (OD) was measured at 492 nm. Pooled sera from *M. tuberculosis* culture-positive patients were used as positive controls (OD, >1.0). Negative control was the antigen coated wells with conjugate and substrate. Antigen-specific responses were expressed as relative titers, which were defined as the optical density of the test specimen minus the optical density of the control wells. Each specimen was run in duplicates in the ALS assay and the mean was used to calculate the results.

In sub-samples with high blood volume (n = 30), cells were incubated and supernatant collected as above to determine the concentration of antibodies in variable cell concentrations (1×10^6^, 2.5×10^6^ and 5×10^6^ cells/ml) against the 6 different TB antigens (Figure 1). In another subsample (n = 30), 5×10^6^ cells/well and 10×10^6^ cells/well were added in smaller volume of culture media (400 microliters/well), incubated for 24 and 48 hours, and culture supernatants collected as above for determination of antibody concentrations. Hereafter this method using low volume of culture media was called the micro-ALS method. The clinical information or the diagnostic report of the patients was not blinded; however laboratory personnel were not aware of the diagnosis during the performance of the ALS test.

### Antigens for coating microtiter plates

The BCG vaccine (freeze-dried, glutamate-BCG vaccine for intradermal use; lot 1861; Japan BCG Laboratories, Japan) used in the Extended Program on Immunization for the vaccination of infants and children in Bangladesh was obtained. CFP10-ESAT6-A, TB51A, TB15.3 were obtained from State Serum Institute, Copenhagen, Denmark; CFP (culture filtrate proteins) and CW (cell wall fraction) from *M. tuberculosis* H37Rv and clinical isolate CSU93, LAM (lipoarabinomannan) of *M. tuberculosis* H37Rv was obtained from Colorado State University, CO, USA (NIH, NIAID Contract NO1 AI-75320). To coat microtiter plates, antigens were used in the following concentrations: BCG vaccine (1 µg/well); LAM (0.01 µg/well); CFP (0.05 µg/well); TB51A (0.05 µg/well); TB15.3 (0.1 µg/well); CW (0.05 µg/well) and fusion protein CFP10-ESAT6-A (1 µg/well). TB51A, TB15.3, CFP-10 and ESAT-6 are proteins that are encoded on the region of difference (RD-1) domain of the *Mycobacterium tuberculosis* genome. Microtiter plates were coated with the different antigens separately/singly and antigen cocktail was not used in any ELISA assay.

### Statistical analysis

Statistical analyses were performed by using PASW (version 17; SPSS Inc., Chicago, IL). Receiver operator characteristic (ROC) analysis was performed to evaluate sensitivity and specificity of different TB antigen specific IgG titers in the ALS assay based on the cutoff of positivity. Positive and negative predictive values, positive and negative likelihood ratio were also determined. Student's t test was applied when comparing between two groups. A P value <0.05 was considered statistically significant.
